# Demographic and practice characteristics of Medicaid-participating dentists

**DOI:** 10.1111/jphd.12037

**Published:** 2013-09-18

**Authors:** Henrietta L. Logan, Yi Guo, Virginia J. Dodd, Christine E. Seleski, Frank Catalanotto

**Affiliations:** 1 Southeast Center for Research to Reduce Disparities in Oral Health, University of Florida, Gainesville, FL, USA; 2 Department of Health Outcomes and Policy, University of Florida, Gainesville, FL, USA; 3 Department of Community Dentistry and Behavioral Science, University of Florida, Gainesville, FL, USA; 4 Department of Biostatistics, University of Florida, Gainesville, FL, USA

**Keywords:** dental care access, children’s dental care, Medicaid, Florida surveys, cultural diversity

## Abstract

**Objectives:**

The challenges entailed in dental Medicaid programs are well documented. To increase our understanding of Medicaid participation, we surveyed Florida dentists to determine the demographic and practice characteristics of Medicaid-participating dentists.

**Methods:**

Our target population was practicing Florida dentists who treat children, including those who do not currently accept Medicaid as well as those who do. The final sample (*n* = 882) included (1) pediatric dentists and (2) general dentists who self-reported that they treat children. Participants completed a survey concerning their Medicaid participation. Analyses included survey-sample weighted chi-square tests and multivariable logistic regression.

**Results:**

More than two-thirds of the sample dentists are not participating in Medicaid and will not consider doing so. Key findings are that Black dentists across the state and Hispanics in South Florida are more likely to participate in Medicaid than other groups of Florida dentists. Pediatric dentists are more likely to be Medicaid participants than general dentists, but nearly one-fifth of the pediatric dentists might quit participation. Non–Medicaid providers are more likely to report not being busy enough in their practice than Medicaid providers.

**Conclusions:**

If we are to address the shortage of Medicaid dental providers, increasing our understanding of how to capture the excess capacity among general dentists (the reported lack of busyness) in a way acceptable to dentists and to the State of Florida is an important first step. In addition, dental schools should consider implementing a track dedicated to training students for practice success within communities of highest dental need and to seek to increase the number of Black dental students.

## Introduction

Oral health is inextricably linked with general well-being, and untreated oral diseases may lead to long-term illnesses and even death (1). The first-ever Surgeon General’s Report on Oral Health was issued in 2000. Terming oral disease a “silent epidemic,” the report posited an association between oral and general health: Oral diseases were linked to ear and sinus infections, a weakened immune system, diabetes, and other serious conditions – and the effect of poor oral health is exacerbated in children. More recently, a study of school performance in children who missed school days because of dental pain or complaints demonstrated that children with poorer oral health status were more likely to perform poorly in school compared to children who missed school for nondental reasons (2). Preventing oral disease may also reduce costs to individuals and society – access to dental care enhances oral health and may be a crucial factor in preventing these costly diseases and their sequelae (3,4).

Medicaid is the largest publicly funded health program for low-income people. It is the principal source of dental care for a majority of low-income children; in 2007, Medicaid covered 29 million children (5). It is estimated that 69 percent of low-income children obtain dental care through government-run safety-net programs, Medicaid, and the State Children’s Health Insurance Program, known in Florida as KidCare. In 2007, the US expenditure on dental services totaled $97 billion, of which $88 billion was derived from private sources – private insurance and out-of-pocket expenses – while Medicaid spent an estimated $7 billion on dental services (6).

Under the Early and Periodic Screening, Diagnostic and Treatment benefit enacted in 1967, provision of dental services is a required service for Medicaid-eligible individuals under the age of 21. According to Centers for Medicaid and Medicare Services guidelines (7), dental services “must include at a minimum, relief of pain and infections, restoration of teeth and maintenance of dental health.” Parents of Medicaid-enrolled children nationwide report difficulty finding dentists who will see them and difficulty getting appointments once they identify a dentist. Many dentists are reluctant to enroll as Medicaid providers – and even dentists who accept Medicaid may restrict the number of Medicaid patients in their practice. According to a report issued by the Department of Health and Human Services (8), about 80 percent of states blamed poor utilization by Medicaid beneficiaries on low participation of dentists in Medicaid; fewer than one in four dentists see more than 100 Medicaid patients in a year (9). More recent data clearly support concerns that low dentist participation in Medicaid persists. In a 2009 Association of State and Territorial Dental Directors survey, most states reported low participation among dentists in state Medicaid programs (10); 25 of 39 states reported that fewer than half of the dentists in these states provided any dental care to Medicaid patients in the prior year. The practice decisions that dentists make every day affect patients’ access to dental care, particularly for the most vulnerable Americans: the poor, the uninsured, and racial and ethnic minorities, including Medicaid recipients. The difficulties of providing care to Medicaid-eligible children are exacerbated in a state like Florida, which has 15 percent of its 18,000,000 residents with incomes below the national poverty level and low levels of dentist participation. Specifically, only 790 dentists, less than 8 percent of practicing dentists in Florida, participate in the dental Medicaid program (11). In 2010, Florida had one of the lowest percentages of Medicaid children enrolled for at least 90 continuous days receiving a preventive dental service (12). To increase our understanding of Medicaid participation, we surveyed Florida dentists to determine the demographic and practice characteristics of Medicaid-participating dentists. In this article, we present the results of a descriptive study that provides demographic and practice characteristics of Medicaid providers and nonproviders in the state of Florida.

## Methods

### Sample

We selected two groups of dentists to participate in this study. First, we selected all pediatric dentists in Florida (*n* = 217) identified in the 2010 directory of the American Academy of Pediatric Dentistry. Second, based on prior work, we identified 2,692 general dentists who self-identified as treating children (11,12). Using preexisting regions (North, Central, and South) established by the Agency for Health Care Administration, we randomly selected 328 general dentists from each region. We sent out 984 surveys to general dentists (self-identified as seeing “kids”), receiving 748 completed surveys, and sent out 217 surveys to pediatric dentists and received 169 complete responses. Thus, the response rate was 76.0 percent for general dentists who self-identified as seeing “kids” and 77.9 percent for pediatric dentists. Of the 917 who responded, 882 (73.4 percent) gave complete responses on the key variable (Medicaid participation) in this report. There was no difference between general dentists and pediatrics in the percentage with complete data on this variable.

### Questionnaire and response

The survey was conducted in English. The rationale for using a single language was that practicing Florida dentists must speak English well enough to successfully pass the licensure exam. The questionnaire was timed to be no more than 20 minutes in length (13,14). To ensure clarity and readability, we pilot-tested the questionnaire on members of the Florida Dental Association (FDA), the Council on Dental Health, the University of Florida Pediatric Dentistry faculty, and second-year pediatric dental residents. The FDA leadership endorsed the survey and methodology and provided a letter of support to be mailed with the survey instrument. We mailed the initial survey via FedEx with a $10 token incentive to encourage response. Participants were also informed that the survey could be accessed via web interface. Data was collected between August 27 and November 3, 2010. The web and paper versions were developed together to be as parallel in visual design and behavior as possible. Survey items and methodology were approved by the University of Florida Institutional Review Board.

### Variables of interest

Medicaid participation was the dichotomous response variable of interest. Dentists were asked, “What statement best describes your current feelings about Medicaid?” The available responses were “not enrolled and will not consider,” “not enrolled but might consider,” “currently enrolled but might drop,” “currently enrolled and will in the future,” and “other.” Individuals who responded that they were “currently enrolled” were classified as Medicaid participants, and all other respondents were classified as nonparticipants.

Ethnicity was divided into two categories: Hispanic and non-Hispanic. Race was divided into three categories: White, Black, and other. Dentists were considered to be of “other” race if they selected Asian, Native Hawaiian or other Pacific Islander, American Indian or Alaska Native, other, or two races or more in the survey. Dentists were asked, “Which of the following best describes your primary practice setting?” Practice type was divided into four categories: solo practice, partner (one of two or more owners), employee (salary, commission, etc.), and other. Given the small number of responses in the categories of “independent contractor,” “owner with associates,” and “other,” a practice was classified as “other” if a dentist selected one of these categories in the survey. Dentists were asked, “How would you best describe your practice during the last 12 months?” Practice busyness was divided into three categories: “too busy,” “busy but not overworked,” and “not busy enough.” Given the small number of dentists who selected“ too busy to accept all appointments” or accepted all appointments but felt overworked, a practice was classified as “too busy” if a dentist selected one of these responses.

### Data analysis

Survey sample weights were created to account for nonresponse and the probability of selection. Differences in practice busyness by characteristics were evaluated using survey-sample weighted chi-square tests. A survey-sample weighted multivariable logistic regression model was used to assess the association of dentists’ Medicaid participation and predictor variables, adjusting for number of years in practice. Predictor variables included gender, race, region, specialty, ethnicity, practice type, and busyness. As the effect of the predictor variables could differ by gender, race, or region, we included in the full model the interactions among these three variables (gender by race, gender by region, race by region) and the interactions of gender, race, and region with the other predictor variables (Table 1). Model selection was performed following strategies described in Muller and Fetterman (15). Starting from the interaction terms, backward step-down model selection was implemented (see Table 1). An exclusion *P* value of 0.05 was used. All analyses were performed using SAS 9.3 (SAS Institute, Cary, NC, USA).

## Results

### Overall characteristics

This report is based on responses from 882 dentists who self-reported that they treat children and identified themselves as either Medicaid participants or Medicaid nonparticipants (663 were men and 219 were women). One hundred sixty-four were pediatric dentists and 718 were general dentists.

As expected, the largest percentage of respondents was in solo practice (74.46 percent), while 16.28 percent reported being in partnerships (see Table 2). There were trends in gender by type of practice, with 76.75 percent of male dentists being in solo practice, followed by the percentage of women who were in solo practice (66.38 percent). There were approximately three times more women working as employee dentists (13.18 percent) than men (4.03 percent). There were differences between pediatric dentists and general dentists in practice type; that is, 60.78 percent of pediatric dentists reported being in solo practice, compared with 75.58 percent of the general dentists. The percentage of pediatric dentists who reported being employees was 13.60 percent, compared with 5.43 percent of general dentists.

### Demographic characteristics by Medicaid participation

Table 3 shows demographic characteristics of dentists who participate in Medicaid and those who do not. The proportion of Hispanics participating in the Medicaid program (32.04 percent) was larger than that not participating (17.92 percent). The proportion of respondents who self-identified as Black participating in Medicaid (13.35 percent) was larger than that not participating (2.46 percent). The proportion of pediatric dentists participating in Medicaid (30.42 percent) was higher than that not participating (4.70 percent). There was a larger proportion of dentists participating in Medicaid in South Florida (54.93 percent) than in either North or Central Florida.

### Practice busyness

Table 4 shows that pediatric dentists reported being significantly busier than general dentists (*P* < 0.0001). There were no significant differences in how busy practices were by region of the state or by gender of the dentist.

### Logistic regression model

We used logistic regression modeling to assess the association of dentists’ Medicaid participation and predictor variables, adjusting for number of years in practice (Table 5). Pediatric dentists were more likely to participate in Medicaid than general dentists [odds ratio (OR) = 11.12; 95 percent confidence interval (CI) = (7.32, 16.9)]. Black dentists were more likely to participate than White dentists [OR = 10.46; 95 percent CI = (4.09, 26.8)]. Dentists who reported they were “busy but not overworked” or “not busy enough” were less likely to participate than dentists who reported they were “too busy,” with ORs of 0.48 [95 percent CI = (0.27, 0.85)] and 0.37 [95 percent CI = (0.19, 0.70)], respectively. There was no significant difference in participation by type of practice, years in practice, or gender between Medicaid participants and nonparticipants. There was a significant interaction between region and ethnicity (*P* = 0.0271). We performed further analysis of the interaction and computed ORs for Hispanic versus non-Hispanic dentists stratified by region. Hispanic dentists in South Florida were more likely to participate than non-Hispanic dentists in South Florida [OR = 3.22; 95 percent CI = (1.49, 6.98)]. A post hoc analysis found that Hispanic dentists in South Florida were more likely to participate than all other ethnicity–region pairings combined [OR = 4.35; 95 percent CI = (2.08, 9.10); *P* < 0.0001].

### Current Medicaid status

**Figure 1:**
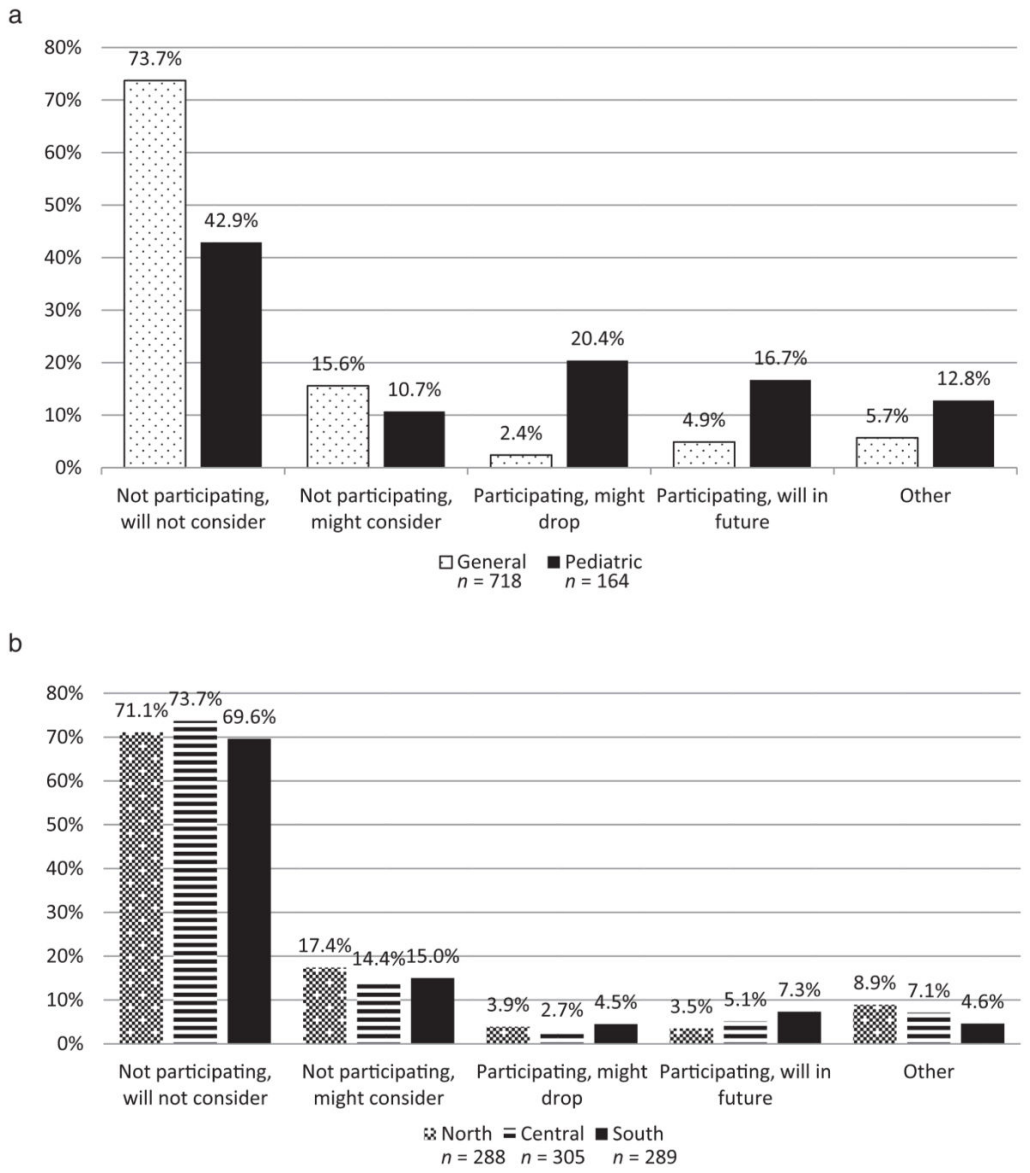
(a) Current Medicaid status stratified by practice type. (b) Current Medicaid status stratified by region. Percentages add up to more than 100% because of some respondents selecting more than one choice. All percentages were calculated using survey-sample weights.

Figure 1a illustrates participants’ current Medicaid status stratified by practice type. Dentists were asked to choose from four options that reflected both their current Medicaid enrollment and the possibility that their Medicaid status might change in the future. More than two-thirds of general dentists (73.7 percent) said that they were not enrolled as a Medicaid provider and would not consider participating, compared with less than half of pediatric dentists (42.9 percent). Of general dentists, 15.6 percent said they were not enrolled but might consider it, compared with 10.7 percent of pediatric dentists. About 7.3 percent of general dentists were currently enrolled, with more than half (4.9 percent of the total) of those saying that they would continue in the future. About 20.4 percent of pediatric dentists said they were participating, but might drop in the future. Another 5.7 percent of general dentists and 12.8 percent of pediatric dentists selected “other” as a choice.

Figure 1b illustrates participants’ current Medicaid status (collapsed by practice type) stratified by region in Florida. In all of the regions, more than two-thirds said they were not enrolled as a Medicaid provider and would not consider participating. About 17.4 percent of dentists in North Florida said they were not enrolled but might consider it, compared with 14.4 percent and 15.0 percent in Central and South Florida, respectively. Approximately 11.8 percent of dentists in South Florida were currently enrolled, with more than half (7.3 percent of the total) of those saying they would continue in the future. Another 8.9 percent, 7.1 percent, and 4.6 percent of dentists in North, Central, and South Florida, respectively, selected “other” as a choice.

## Discussion

Key findings from this study include that Blacks across the state and Hispanics in South Florida were more likely to treat Medicaid patients than other groups of Florida dentists. More than two-thirds of the total sample were not participating in Medicaid and would not consider doing so. In addition, nearly one-fifth of the Medicaid-participating pediatric dentists said they might drop in the future. Non–Medicaid providers were more likely to report not being busy enough in their practice than Medicaid providers.

This is one of only a few studies providing detailed characteristics of dentists who participate in Medicaid and those who do not. These findings are instructive in understanding how age, race, sex, geographical location, and practice characteristics influence a dentist’s disposition in regard to Medicaid participation and planning interventions. We recognize that one of the limitations of this study is that dentists self-reported whether they saw children, and we are unsure of the number of children seen and the age definition of “children.” We also do not know how many children these self-identified dentists see in a year or the age threshold at which the dentists treat.

Black dentists were more likely to be Medicaid providers, which might indicate a higher concern for underprivileged populations among this group of dentists. This finding is similar to those reported from Wisconsin, in which racial/ethnic-minority dentists were twice as likely as White dentists to accept new Medicaid patients (16). Studies of other medical professions have also found that minority practitioners were more likely to treat Medicaid patients (17). That being said, greater representation of Blacks in the dental profession and in dental schools may be a way to increase the number of Medicaid providers (18–20). Although we recognize that greater enrollment of Blacks in dental schools does not guarantee greater participation in Medicaid, prior research points in that direction (16). On the other hand, the data on dentists of Hispanic ethnicity by region show that Hispanics in South Florida were more likely to accept Medicaid, and post hoc analysis showed that dentists in this group were more likely to be Medicaid participants than those in all other groups combined. However, Hispanics in Central and North Florida were no more likely to be Medicaid providers than non-Hispanic dentists.

One of the more surprising findings was that dentists who reported they were “not busy enough” or “busy but not overworked” were less likely to participate as Medicaid providers than dentists who reported they were “too busy.” The reasons these dentists do not accept Medicaid to fill their chairs is unclear but certainly warrants additional consideration.

The observation that pediatric dentists had a much higher rate of enrollment than general dentists is not surprising. Comprehensive Medicaid dental benefits for children are mandated nationally and in Florida, while optional adult benefits in the state are essentially limited to extractions and dentures. Most pediatric dental practices are physically equipped (multichair dental operatories) to provide care to large numbers of children, whereas adults expect the privacy of an individual operatory. Therefore, pediatric dentists may be better able to fiscally manage the relatively low Medicaid reimbursement rates. In addition, many general dentists have expressed inadequacies about their ability to manage very young children (21–23). On the other hand, the finding that nearly one-fifth of the Medicaid-participating pediatric dentists might discontinue participation is a great concern and warrants attention at the highest level. Pediatric dentists are more likely to provide preventive care than are other dentists; fewer pediatric dentists seeing children in Medicaid is likely to have serious consequences (23,24).

A recent report by the Florida Public Health Institute indicated that in 2010, 115,000 patients in Florida utilized hospital emergency rooms (ERs) for preventable dental problems. Of those patients, 40,430 patients (adults and children) covered by Medicaid were seen in the ER at a cost of $29,751,245 (based on Medicaid reimbursement fees) (25).

These data about ER use for preventable dental problems further substantiate the importance of improving the Medicaid dental program in Florida. This point, coupled with our finding that non–Medicaid participants reported being significantly less busy than Medicaid providers, poses an interesting possibility. If there is greater provider capacity to treat Medicaid enrollees in Florida, then efforts should be made to associate this greater capacity for care with those individuals using the ER for preventable dental problems. The resulting cost reduction to the state might translate into ways to incentivize caregivers and patients toward greater use of preventive dental services that are provided in the dental office.

## Recommendations

Florida represents a broad cross-section of both dental practitioners and the communities they serve. The high response rate to this survey indicates that dentist-respondents in the state of Florida felt the issue was relevant and important to their practice. A task force to study ways to capture the excess capacity among general dentists (lack of busyness) in a way acceptable to dentists and to the State of Florida seems an important next step in resolving the two horns of the current dental care dilemma: lack of access and unused capacity. In addition, the inclusion of a dedicated track in dental education that focuses on the care of children and the operation and economics of an urban (or rural) health center should be strongly considered by each dental school and at the national leadership level. Such a track should be dedicated to training students for success – financially and professionally – within communities of the highest need. Finally, to address the short-age of Medicaid dental providers, dental schools should seek to increase the number of Black practitioners by enrolling larger numbers of Blacks as students, as these data suggest that Black dentists are the most likely to be Medicaid providers.

## Figures and Tables

**Table 1:** Variables in Initial Regression Model

Variable Category	Variables
Covariate	Years in practice
Predictors of Interest	Gender, race, region specialty, ethnicity, practice type, busyness
Interactions	Gender × race, gender × region, race × region, Gender × (specialty, ethnicity, practice type, busyness), Race × (specialty, ethnicity, practice type, busyness), Region × (specialty, ethnicity, practice type, busyness)

For an explanation of the initial model, see “Data Analysis” subsection of Methods. Backward step-down model selection proceeded from the bottom of the table to the top. Interactions were tested as “chunks” one row at a time. Statistically significant “steps” were broken down with each two-way interaction term tested individually.

**Table 2:** Overall Characteristics of Dentists by Practice Type

	Solo practice (%)	Partnership (%)	Employee (%)	Independent contractor (%)	Owner with associates (volunteered) (%)
All (*n* = 882)	74.46	16.28	6.05	1.53	1.68
Gender					
Male (*n* = 663)	76.75	15.84	4.03	1.46	1.92
Female (*n* = 219)	66.38	17.87	13.18	1.75	0.82
Specialty					
Pediatric (*n* = 164)	60.78	23.63	13.60	0.66	1.33
General (*n* = 718)	75.58	15.69	5.43	1.60	1.70

All percentages were calculated using survey-sample weights.

**Table 3:** Demographic Characteristics of Medicaid Participants and Non-participants

	Participants *n* = 126	Non-participants *n* = 756
Mean age (years)	50.49 ± 12.39	52.11 ± 9.26
Mean years in practice	22.81 ± 12.95	24.91 ± 9.67
Gender		
Male	63.38%	79.73%
Female	36.62%	20.27%
Ethnicity		
Hispanic	32.04%	17.92%
Non-Hispanic	67.96%	82.09%
Race		
White	71.40%	84.92%
Black	13.35%	2.46%
Other	15.25%	12.62%
Specialty		
Pediatric	30.42%	4.70%
General	69.58%	95.30%
Region		
North	14.04%	18.28%
Central	31.03%	36.11%
South	54.93%	45.61%
Type of practice		
Solo	72.93%	74.65%
Partner	12.82%	16.71%
Employee	11.11%	5.43%
Other	3.14%	3.21%

All percentages were calculated using survey-sample weights.

**Table 4:** Practice Busyness by Characteristics

	Too busy to accept all appointments (%)	Accepted all appointments but felt overworked (%)	Accepted all appointments, was not overworked (%)	Not busy enough (%)	*P* value
Region					
North (*n* = 288)	3.24	16.91	51.12	28.73	0.1142
Central (*n* = 305)	3.91	13.00	48.43	34.66	
South (*n* = 289)	0.95	13.75	50.49	34.81	
Gender					
Male (*n* = 663)	2.39	13.19	49.58	34.84	0.4732
Female (*n* = 219)	2.47	17.07	50.90	29.56	
Specialty					
Pediatric (*n* = 164)	5.52	16.50	61.63	16.36	0.0001
General (*n* = 718)	2.16	13.85	48.92	35.08	

All percentages were calculated using survey-sample weights.

**Table 5:** Results from Logistic Regression Model

Variable	Estimate (SE)	Odds ratio (95% CI)	*P* value
Years in practice	0.10 (0.14)	1.11 (0.84, 1.46)	0.4701
Gender			
Male	−0.22 (0.30)	0.80 (0.45, 1.43)	0.4561
Female	Reference	Reference	Reference
Specialty			
Pediatric	2.41 (0.21)	11.12 (7.32, 16.9)	<0.0001
General	Reference	Reference	Reference
Region			
North	Reference	Reference	Reference
Central	−0.03 (0.30)	0.97 (0.54, 1.76)	0.9299
South	−0.15 (0.28)	0.86 (0.50, 1.49)	0.5928
Ethnicity			
Hispanic	1.17 (0.39)	3.22 (1.49, 6.98)	0.0030
Non-Hispanic	Reference	Reference	Reference
Race			
White	Reference	Reference	Reference
Black	2.35 (0.48)	10.46 (4.09, 26.8)	<0.0001
Other	0.39 (0.34)	1.48 (0.76, 2.85)	0.2467
Type of practice			
Solo	Reference	Reference	Reference
Partner	−0.40 (0.34)	0.67 (0.35, 1.29)	0.2290
Employee	0.03 (0.52)	1.03 (0.37, 2.88)	0.9541
Other	0.15 (0.73)	1.16 (0.28, 4.90)	0.8386
Practice busyness			
Too busy	Reference	Reference	Reference
Busy but not overworked	−0.74 (0.29)	0.48 (0.27, 0.85)	0.0119
Not busy enough	−1.00 (0.33)	0.37 (0.19, 0.70)	0.0022
Ethnicity × region			0.0271
Non-Hispanic	Reference	Reference	Reference
Hispanic in South	1.17 (0.39)	3.22 (1.49, 6.98)	0.0030
Hispanic in Central	−0.44 (0.61)	0.65 (0.20, 2.13)	0.4732
Hispanic in North	0.77 (0.88)	0.46 (0.08, 2.60)	0.3806

SE, standard error; CI, confidence interval.
